# Effects of a 4-week plyometric training on activity patterns during different phases of one-leg drop jump with focus on jump height

**DOI:** 10.1038/s41598-023-36461-1

**Published:** 2023-06-06

**Authors:** Somayeh Ahmadabadi, Hamid Rjabi, Reza Gharakhanlou, Saeed Talebian, Aref Basereh

**Affiliations:** 1 Department of Physical Education and Sports Sciences, Farhangian University, Tehran, Iran; 2 Department of Exercise Physiology, Faculty of Physical Education and Sports Sciences, Kharazmi University, Tehran, Iran; 3 Department of Physical Education and Sports Sciences, Faculty of Humanities, Tarbiat Modares University, Tehran, Iran; 4 Department of Physiotherapy, School of Rehabilitation, Tehran University of Medical Sciences (TUMS), Tehran, Iran; 5 Department of Physical Education and Sports Sciences, Kharazmi University, Tehran, Iran

**Keywords:** Musculoskeletal system, Ligaments, Muscle, Motor neuron, Neuromuscular junction

## Abstract

Athletic women have shown a higher risk of ACL injury during jump landing compared to men. Plyometric training can be an alternative way to minimize the risk of knee injuries via the changed muscle activity patterns. Hence, the aim of this study was to determine the effects of a 4-week plyometric training program on the muscle activity pattern in different phases of one-leg drop jump in active girls. Active girls were randomly allocated into 2 groups (Plyometric training = 10, Control group = 10) where the plyometric training group (PTG) performed 60 min exercises, 2 sessions/1 week for 4 weeks while the control group (CG) had their daily activity. In the pre to post test, the sEMG was recorded from the Rectus Femoris (RF), Biceps Femoris (BF), Medial Gastrocnemius (GaM), and Tibialis Anterior (TA) muscles of the dominant leg during the Preparatory phase (PP), Contact Phase (CP), Flight Phase (FP) of one-leg drop jump. Electromyography variables (Signal amplitude, Maximum activity, Time to peak (TTP), Onset and activity time and Order muscle activity) and Ergo jump variables (Time of preparatory phase (TPP), Time of contact phase (TCP), Time of flight (jump height) phase (TFP), and Explosive power were analyzed. The Univariate ANCOVA test showed a significant difference between the two groups in Activity Time, whilst adjusting for pre-test as a Covariate, only in TA muscle (F_(1,17)_ = 5.09, p = 0.038, η^2^ = 0.230). In PTG. TA (− 15%), GaM (− 19%), and BF muscles (− 9%) started their activity earlier while there was no significant difference between the two groups at the Onset time. TTP of RF was significantly different between the 2 groups only in the PR phase (0.216 ± 0.07 vs 0.153 ± 0.09 s) (p = 0.049, 95% CI = 0.001, 0.127). Results of the present study suggest that a 4-week plyometric training can improve the stability of leg joints via earlier recruitment of muscles and change activity patterns in lower limb muscles. It also recommends that the preparatory phase before landing be considered an important stage in preventing sports injuries in a training program.

## Introduction

Plyometric training is generally expected to enhance performances during stretch–shortening cycle (SSC) exercises. Among the mechanisms of plyometric training, it is possible to mention the potential of the electric currents of the muscle (neural adaptations), biochemical-mechanical potential, and restoring the elastic energy of the muscles^1,2^. Plyometric training causes modulation in the action of deep muscle receptors. Studies have shown that the muscle spindle participates in power output through a stretch reflex mechanism in plyometric training^3^. By contrast, another proprioceptor, Golgi tendon organs (GTOs), located in tendons, mainly prevents muscle over-contraction^3^, which reduces the sensitivity of this organ to plyometric training^4^, which increases the recruitment of motor units and changes the pattern of muscle activity during jumping^5,6^. Therefore, plyometric training seems to increase the neuromuscular control of the knee joint during landing due to pre-preparation, which reduces knee joint injuries^7,8^. Typically, plyometric training is carried out over a period of several days or weeks (6–12 weeks), at a training frequency of 1–3 sessions per week, and a maximal to near-maximal intensity^9^. On the other hand, Studies indicate that short-duration plyometric training (4 weeks) can also improve jump and movement performance^10–14^.

The most important factor in controlling movement in the knee is the responsibility of the quadriceps and hamstring muscles, and due to the importance of the flexion mechanism, the function of the quadriceps muscles is very important in the movement control process with concentric and eccentric contractions^15^. Studies have shown that the rectus femoris muscle is the main responsible for holding the knee joint against additional loads^16^. Several studies have also shown that knee and thigh angles during landing are essential determinants of forces on the knee. In other words, small flexion angles produce strong impact forces^17,18^. In this context, Reimann showed that a successful landing from a jump requires co-activation of the hamstring and quadriceps muscle groups, increasing muscle stiffness and, as a result, joint stability in the lower body^19^. Single-leg landing is a common task in sports that requires a sudden stop and a quick change of direction. The single-legged DJ has a more beneficial training effect than the double-legged DJ^20,21^. Wang et al. measured the jumping height, ground contact time, reactive strength index, ground reaction force, ground reaction force loading rate, joint power, and stiffness in 12 male college students during the single-legged and double-legged DJ and their results showed that the single-leg was more effective than the double-leg in all variables, especially when the drop height of the single-legged DJ was less than 30-cm^20^. However, limited research has investigated the activity pattern of lower body muscles in jumping and landing on one leg after plyometric training, these studies compare successful and unsuccessful landings on the domain and non-domain leg^22^, investigating the factors causing injuries to the anterior cruciate ligament (ACL)^23^, the comparison of depth jump of healthy people with functional instability^24^ and gender differences in lower body kinematics during single-leg landing^25^ have been discussed. Various studies were conducted in relation to plyometric training and its beneficial effects on preventing damage to lower body organs and improving strength performance in both genders^17^. These studies showed that females demonstrate a lower degree of knee flexion and a greater degree of thigh extension than men in all sports^26^. The females tend to drop into a more vertical position with less knee and hip flexion, tibial rotation, and abnormal knee external rotation^27^. Also, female athletes showed an increase in the activity of the quadriceps compared to their opposite muscle group (hamstring) in landings^27^.

The increased homogeneity in muscle activity at the moment of landing begins before the foot contact and provides greater protection and stability of the joint against damage^28^. Therefore, knowing the pattern of muscle activity during different stages of drop jump helps to understand the mechanisms of increasing mechanical efficiency during the stretching-shortening cycle. Hence, in this research, we have two specific hypotheses included: (1) 4 weeks of plyometric training can change EMG variables of different muscles (Rectus Femoris, Biceps Femoris, Tibialis Anterior, and Medial Gastrocnemius) in different phases (preparatory, contact, jump phases) of a one-leg drop jump in active girls, (2) 4 weeks of plyometric training can change Ergo-jump variables of different muscles (Rectus Femoris, Biceps Femoris, Tibialis Anterior, and Medial Gastrocnemius) in different phases (preparatory, contact, jump phases) of a one-leg drop jump in active girls.

## Materials and methods

### Participants

The G power 3.0.10 program was used to calculate the minimal sample size needed in our study, with Z1-β = 1.03 (power = 85%) and *Z*/2 = 1.96 (α = 5%), and with considering a ratio of 1 control for every case, there was a need for a minimum of 10 experimental and 10 control subjects. Subjects were recruited via advertisements, which were posted on bulletin boards at a university and a research institute; telephone calls, which were made to individuals on the institute’s volunteer registry; and word-of-mouth. Individuals were deemed eligible to participate in this study if they: (a) were available to attend all assessment and intervention sessions; (b) and free from any known history of neuromuscular impairment, or musculoskeletal injuries over the recent 6 months. Twenty participants volunteered to participate in the study. Participants assigned to the PT group were required to undertake 8 supervised plyometric training sessions over a 4-week training period. Participants assigned to the control group were not completed any training sessions. Participants’ characteristics are detailed in Table 1.

**Table 1:** Initial characteristics of experimental and control groups.

	Experimental group (*n* = 10)	Control group (*n* = 10)
Age (years)	21.8 ± 0.63	21.5 ± 0.97
Height (m)	1.65 ± 0.05	1.63 ± 0.05
Body mass (kg)	57.33 ± 8.84	56.65 ± 10.39
BMI (kg/m^2^)	21.1 ± 2.5	21.3 ± 1.9
Fat percent (%)	29.32 ± 6.7	27.84 ± 8.53

The initial test measurements included height measurements (using a Seka model 222 made in Ireland), age, weight, fat percentage (using an Omron model HBF-508 scale), and determination of the domain leg. 3 tests were used to determine the dominant leg: ball hitting test^22^, step climbing test, and balance-rest test^29^. The leg that was used in at least 2 tests was determined as the domain leg.

### Procedures

The study examined the impact of a 4-week PT program (two sessions per week, with a 48-h rest interval) on muscle activity patterns (Rectus Femoris, Biceps Femoris, Tibialis Anterior, Medial Gastrocnemius) in different phases (preparatory, contact, jump) during a one-leg drop jump. All protocols were approved by the Tarbiat Modares University Research Committee and Review Board in Physical Education Department. This study was conducted according to the ethical principles of the Declaration of Helsinki and approved by the Institutional Review Board of Tarbiat Modares University (#TMU/D/10901181390). Written informed consent forms were signed by all participants.

The subjects of the experimental group performed the plyometric training for 4 weeks, 2 sessions each week, a total of 8 sessions (Table 2). Participants were allocated into the control group were not completed any training sessions. In the design of the main training program, the increasing load intensity is adjusted based on increasing the height of the step. In each session, all members of the experimental group attended the training place at the same time and the plyometric training program was performed under the supervision of two experienced coaches and with the coordination of the research team leader. According to the research, the pattern of muscle activity changes with increasing the height of the step from 40 to 60 or 80 cm, and not changes lower than 40 cm^30^. Therefore, to perform the pre and post-test, a single-leg drop jump was used from a step with a height of 20 cm^29^. The schedule of each plyometric training session included three parts: warm-up (15 min), Training (45 min), and cool-down (15 min). The important point related to the plyometric training program in this study was that the training was performed bilaterally with both legs in different directions while the test (pre and post) was done unilaterally (with the domain leg). For every 3 s of contraction, 30 s of rest were considered^31^. The subjects were asked to do their maximum effort in each movement.

**Table 2:** 4-week plyometric training program.

	Weeks			
	1	2	3	4
Height of the step	10 cm	15 cm	20 cm	25 cm
Movement				
Hop right foot (10 m)	2	2	2	2
Hop left foot (10 m)	2	2	2	2
One-legged vertical jump in front of the step with a change of legs	2 × 10	2 × 10	2 × 10	2 × 10
Jumping with the right leg to the front step	2 × 10	2 × 10	2 × 10	2 × 10
Jumping with the left leg to the front step	2 × 10	2 × 10	2 × 10	2 × 10
Jumping with the right leg to the side of the step	2 × 10	2 × 10	2 × 10	2 × 10
Jumping with the left leg to the side of the step	2 × 10	2 × 10	2 × 10	2 × 10
Jumping with the right leg to the back of the step	2 × 10	2 × 10	2 × 10	2 × 10
Jumping with the left leg to the back of the step	2 × 10	2 × 10	2 × 10	2 × 10

### Pre and post measurements

**Figure 1:**
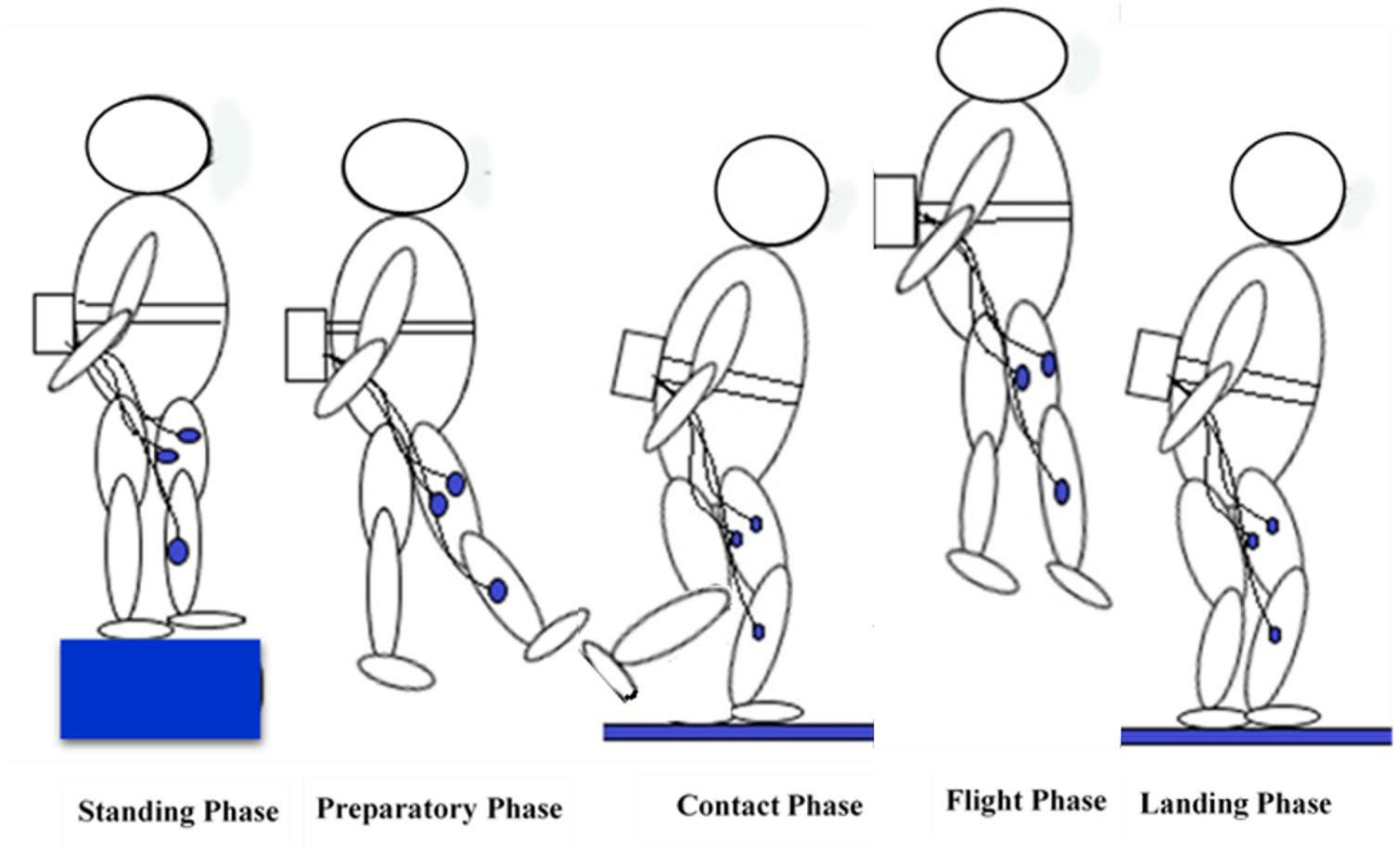
Drop jump phases.

For pre and post-test measurements, the participants standing on the step, hearing the sound stimulus, without any contraction in the leg muscles, let themselves down from the steps, and as soon as the domain leg touched the ground, immediately jumped up and then landed on the floor. Also, for the skill to be performed in a completely natural way, the subject's hands were opened, and asked not to use their hands to perform the jump and to put them on their waist^32^ (Fig. 1). Also, to perform the drop jump skills uniformly, the subjects should be asked not to flexion their knees during the jump-up phase. In order to study the pattern of muscle activity during this drop jump, the data was recorded by electromyography and Ergo-jump. Each drop jump was divided into phases: standing on the platform, Preparatory phase, contact phase, Flight phase, and land phase. Preparatory, Contact, and Flight phases have been evaluated. After recording EMG and Ergo-jump data and according to the results of 3 jump repetitions, the repetition with the highest flight time (jump height)^33^ was selected as the main jump, and Ergo-jump and EMG data analysis was performed on it.

### Electromyographic (EMG) assessment

The area of electrode placement was shaven to remove fine hair, rubbed with an abrasive skin gel to remove dead skin, and then cleaned with 70% isopropyl alcohol wipes. The sEMG was recorded from the Rectus Femoris (RF), Biceps Femoris (BF), Medial Gastrocnemius (GaM), and Tibialis Anterior (TA) muscles of the dominant leg using bipolar Ag–AgCl electrodes (SKINTACT, made German). The sEMG signals were amplified (× 1000), bandpass filtered (20 Hz–1 kHz), digitized online at 2 kHz, recorded (ME6000 device), and analyzed using MegaWin software (v.3.1). Variables from EMG: onset time, duration time of activity, maximum activity, signal amplitude, and time to peak. The analysis of the sEMG signal was done in such a way that at first the RMS of the raw signal was calculated and then it was normalized by using the averaging window 51 and the amplitude of the RMS signal using the formula (amplitude of each phase/resting amplitude signal).

### Ergo-jump assessment

The Ergojump (manufactured by Danesh Salar Iranian, Iran) had 2 plates. One of them was placed on the step and the other was on the ground. Variables calculated by Ergo-jump device^34^: Time of preparatory phase (TPP), Time of contact phase (TCP), Time of flight (jump height) phase (TFP), and Explosive power. For measuring the Time of the Preparatory phase, subjects were asked to drop themselves without any lower limb contraction from the step on the first plate. The time between this drop and the initial contact of the foot with the second plate on the floor was TPP. The total time of first contact with the second plate was recorded as TCP. As soon as the subject contacted the second floor, they were asked to jump. This time between the jump and second contact was recorded as the duration of the flight time (Fig. 1). Also, the average explosive power is calculated using Bosco's formula (W = (Tf × Tt × g2)/4n (Tt − Tf)) with the unit (W/kg). In this formula, Tf: flight time (s), Tt: test duration (s), n: number of jumps, and g = 9.8^35^.

### Statistical analysis

Descriptive statistics were used to summarize the characteristics of the participants and measurements. The normality of data was assessed with the Shapiro–Wilk test. ANCOVA (Pre-test as a covariate) followed by Bonferroni post hoc test was used to evaluate the effects of a 4-week plyometric training on activity pattern variables of 4 muscles in different phases of a drop jump. Bonferroni's post hoc test was performed to evaluate the differences within a group. All statistical analysis was performed using Spss V22. A p < 0.05 was considered for statistical significance.

### Ethics approval

All protocols of this research were approved by the Tarbiat Modares University Research Committee and Review Board. This study was conducted according to the ethical principles of the Declaration of Helsinki, and approved by the Institutional Review Board of the Tarbiat Modares University, Department of Physical Education (#TMU/T/10901181390).

### Patient consent statement

All the participants completed and signed informed consent.

## Results

### EMG variables

#### Signal amplitude

The electromyography results of the lower limbs are shown in Table 3. The results of the Univariate ANCOVA test showed no significant difference between the two groups in signal amplitude in the different Phases (Phases × Group) of jumping, whilst adjusting for pre-test, in all muscles (p > 0.05).

**Table 3:** Electromyography results for control group and plyometric group (mean ± SD).

Muscle	Variable	Phases	Control group		Plyometric group		Univariate ANCOVA							
			Pre-test	Post-test	Pre-test	Post-test	Pre-test		Phases		Group		Phases × Group	
							F	p	F	p	F	p	F	p
Rector femoris	Signal amplitude (μv)	PR	10.73 ± 12.00	5.34 ± 5.36	4.80 ± 6.58	5.94 ± 6.25	0.46	0.49	3.86	0.03*	0.04	0.84	0.18	0.84
		CO	26.75 ± 25.70	13.11 ± 7.10	9.88 ± 8.60	12.50 ± 14.63								
		FL	36.41 ± 30.82	19.61 ± 9.31	13.82 ± 10.55	18.82 ± 24.33								
	Maximum activity (μv)	PR	56.1 ± 38.82	64.9 ± 50.83	55.80 ± 40.86	57.80 ± 41.02	8.84	0.000**	1.32	0.27	0.94	0.33	0.41	0.66
		CO	170.6 ± 111.63	164.5 ± 127.99	152.0 ± 97.59	141.1 ± 13.09								
		FL	196.1 ± 118.49	241.2 ± 191.54	183.6 ± 95.60	166.6 ± 19.08								
	Time to peak (s)	PR	0.194 ± 0.10	0.216 ± 0.07	0.187 ± 0.10	0.153 ± 0.09	20.02	0.16	5.9	0.000**	0.17	0.68	3.73	0.03*
		CO	0.231 ± 0.05	0.213 ± 0.07	0.233 ± 0.09	0.268 ± 0.07								
		FL	0.169 ± 0.08	0.135 ± 0.07	0.221 ± 0.07	0.176 ± 0.07								
Biceps femoris	Signal amplitude (μv)	PR	2.44 ± 1.80	4.10 ± 4.1	2.65 ± 2.16	3.1 ± 1.74	3.11	0.08	3.99	0.000**	7.75	0.000**	1.24	0.29
		CO	6.82 ± 3.59	10.98 ± 7.00	5.73 ± 4.00	6.27 ± 4.13								
		FL	10.00 ± 5.45	15.41 ± 9.48	8.0 ± 5.37	8.0 ± 4.30								
	Maximum activity (μv)	PR	46.0 ± 42.35	51.5 ± 40.77	46.0 ± 29.97	59.1 ± 41.81	3.93	0.05*	12.39	0.000**	1.64	0.20	0.92	0.41
		CO	100.1 ± 48.76	118.7 ± 45.40	79.6 ± 34.13	99.2 ± 52.68								
		FL	119.0 ± 60.13	131.9 ± 51.90	103.1 ± 41.88	107.3 ± 54.91								
	Time to peak (s)	PR	0.228 ± 0.05	0.216 ± 0.06	0.201 ± 0.07	0.165 ± 0.10	3.41	0.07	4.35	0.01*	0.28	0.59	1.43	0.24
		CO	0.233 ± 0.05	0.207 ± 0.09	0.247 ± 0.08	0.25 ± 0.08								
		FL	0.142 ± 0.10	0.133 ± 0.09	0.199 ± 0.09	0.119 ± 0.09								
Tibialis anterior	Signal amplitude (μv)	PR	11.24 ± 7.00	13.77 ± 15.19	5.97 ± 7.73	4.45 ± 2.66	10.93	0.000**	0.18	0.83	4.90	0.03*	0.20	0.82
		CO	16.28 ± 12.78	13.36 ± 12.28	6.0 ± 8.0	12.50 ± 14.63								
		FL	16.94 ± 15.0	12.76 ± 10.12	4.34 ± 3.16	4.24 ± 4.69								
	Maximum activity (μv)	PR	216.3 ± 110.36	169.5 ± 92.44	215.2 ± 15.8	164.1 ± 134.0	16.05	0.000**	0.05	0.95	0.73	0.39	0.12	0.89
		CO	259.9 ± 105.35	210.6 ± 113.35	212.4 ± 151.7	160.6 ± 115.4								
		FL	246.6 ± 88.16	201.2 ± 130.72	167.9 ± 108.9	133.0 ± 78.08								
	Time to peak (s)	PR	0.199 ± 0.09	0.184 ± 0.09	0.183 ± 0.06	0.117 ± 0.10	1.7	0.25	0.42	0.65	0.05	0.83	1.33	0.27
		CO	0.200 ± 0.10	0.115 ± 0.12	0.085 ± 0.10	0.121 ± 0.10								
		FL	0.064 ± 0.08	0.086 ± 0.10	0.149 ± 0.10	0.105 ± 0.08								
Medial gastrocnemius	Signal amplitude (μv)	PR	7.87 ± 14.4	4.61 ± 8.9	1.04 ± 0.55	1.66 ± 0.66	5.32	0.03*	0.80	0.45	0.65	0.42	0.04	0.96
		CO	13.48 ± 17.61	8.51 ± 12.37	2.49 ± 1.42	3.99 ± 3.49								
		FL	14.82 ± 14.89	10.45 ± 14.63	3.38 ± 2.0	4.93 ± 4.29								
	Maximum activity (μv)	PR	105.0 ± 63.01	205.7 ± 167.8	127.5 ± 122.3	147.8 ± 118.9	0.34	0.56	5.58	0.000**	5.56	0.02*	0.18	0.83
		CO	215.7 ± 83.12	342.6 ± 145.89	236.1 ± 175.3	245.6 ± 121.0								
		FL	243.5 ± 105.07	374.2 ± 159.28	256.5 ± 188.6	265.4 ± 121.5								
	Time to peak (s)	PR	0.154 ± 0.10	0.185 ± 0.09	0.155 ± 0.10	0.151 ± 0.11	0.56	0.46	4.21	0.02*	0.03	0.86	0.38	0.68
		CO	0.141 ± 0.04	0.230 ± 0.07	0.250 ± 0.08	0.250 ± 0.10								
		FL	0.141 ± 0.10	0.136 ± 0.11	0.214 ± 0.05	0.150 ± 0.10								

*P < 0.05, **P < 0.01.

Also, the results of the Univariate ANCOVA test of signal amplitude showed a significant difference between phases of jumping in RF (p = 0.03, η^2^ = 0.132) and BF muscles (p < 0.001, η^2^ = 0.131). Bonferroni's post hoc test showed a significant difference between the PR Phase compared to FL Phase in the signal amplitude of RF muscle (p < 0.001, 95% CI = 1.61, 23.64) and BF muscle (p < 0.001, 95% CI = -2.37, 6.73).

#### Maximum activity

The results of the Univariate ANCOVA test showed no significant difference between the two groups in Maximum activity in the different Phases (Phases × Group) of jumping, whilst adjusting for pre-test, in all muscles (p > 0.05).

Also, the results of the Univariate ANCOVA test showed a significant difference in the phases of jumping in BF (p < 0.001, η^2^ = 0.319) and GaM muscles (p < 0.001, η^2^ = 0.176). Bonferroni's post hoc test for BF muscle showed a significant difference in PR Phase compared to FL Phase (p < 0.001, 95% CI = 39.517, 126.57) and CO phase (p < 0.001, 95% CI = 26.32, 106.24). For GaM muscle post hoc test showed a significant difference in PR phase compared to FL Phase (p < 0.001, 95% CI = 33.87, 275.23) and CO Phase (p < 0.001, 95% CI = 9.33, 244.22).

#### Time to peak (TTP)

The results of the Univariate ANCOVA test showed a significant difference in the time-to-peak variable between the two groups in the different phases (Phases × Group) of jumping, whilst adjusting for pre-test, only in the time-to-peak variable in the RF muscle (F_(2,53)_ = 3.72, p = 0.03, η^2^ = 0.319). Other muscles did not show a significant difference between the two groups in the different phases (Phases × Group) of jumping (p > 0.05). Bonferroni's post hoc test for RF muscle showed that there was a difference between the two groups only in the PR phase of the jumping phase (0.216 ± 0.07 vs 0.153 ± 0.09 s) (p = 0.049, 95% CI = 0.001, 0.127).

Also, the results of the Univariate ANCOVA test showed a significant difference in the phases of jumping in RF (p < 0.001, η^2^ = 0.182), BF (p = 0.01, η^2^ = 0.141) and GaM muscles (p = 0.02, η^2^ = 0.137). Bonferroni's post hoc test for RF muscle showed a significant difference in CO Phase compared to FL phase (p = 0.004, 95% CI = 0.22, 0.126). For BF muscle post hoc test showed a significant difference in CO phase compared to FL phase (p = 0.015, 95% CI = 0.013, 0.153). For GaM muscle post hoc test showed a significant difference in CO phase compared to FL phase (p = 0.018, 95% CI = 0.018, 0.169).

#### Onset and activity time and order muscle activity

The results of the Univariate ANCOVA test showed no significant difference between the two groups in Onset time, whilst adjusting for pre-test, in all muscles (p > 0.05).

The results of the Univariate ANCOVA test showed a significant difference between the two groups in Activity Time, whilst adjusting for pre-test, only in TA muscle (F_(1,17)_ = 5.09, p = 0.038, η^2^ = 0.230), Other muscles did not show a significant difference between the two groups (p > 0.05). Bonferroni's post hoc test for TA muscle showed that the duration of Activity Time in the plyometric training group was longer than the control group (1.02 ± 0.24 vs 1.25 ± 0.28 s) (p = 0.038, 95% CI = 0.017, 0.516).

Table 4 shows Order muscle activity patterns are different between the two groups. In the training group, the TA and RF muscle followed the same muscle activity pattern from pre to post-test, but the BF and the GaM muscle changed the activity pattern from pre to post-test.

**Table 4:** Onset and activity time and muscle activity pattern for control group and plyometric group (mean ± SD). Significant values are in bold.

Muscle	Variable	Control group		Plyometric group		Univariate ANCOVA			
						Pre-test		Group	
		Pre-test	Post-test	Pre-test	Post-test	F	p	F	p
Rectus femoris	Onset time (s)	2.11 ± 0.72	1.85 ± 0.78	1.73 ± 0.66	1.42 ± 0.47	1.32	0.26	1.0	0.27
	Activity time (s)	1.19 ± 0.49	1.23 ± 0.49	1.21 ± 0.34	1.18 ± 0.26	4.03	0.06	0.12	0.74
	Order muscle activity pattern	2	1	2	2				
Biceps femoris	Onset time (s)	2.43 ± 0.67	2.27 ± 0.85	1.80 ± 0.6	1.64 ± 0.4	0.16	0.69	2.17	0.12
	Activity time (s)	0.8 ± 0.27	0.89 ± 0.22	1.13 ± 0.53	0.95 ± 0.22	5.41	0.03	0.05	0.82
	Order muscle activity pattern	4	4	3	4				
Tibialis anterior	Onset time (s)	1.99 ± 0.62	1.95 ± 0.82	1.57 ± 0.61	1.34 ± 0.39	0.01	0.91	3.83	0.06
	Activity time (s)	1.4 ± 0.48	1.02 ± 0.24	1.26 ± 0.33	1.25 ± 0.28	1.53	0.23	5.09	0.03*
	Order muscle activity pattern	1	2	1	1				
Medial gastrocnemius	Onset time (s)	2.17 ± 0.64	1.99 ± 0.79	1.88 ± 0.95	1.52 ± 0.59	0.27	0.61	1.90	0.18
	Activity time (s)	1.29 ± 0.07	0.93 ± 0.53	1.06 ± 0.5	1.08 ± 0.5	0.15	0.69	0.50	0.48
	Order muscle activity pattern	3	3	4	3				

*P < 0.05.

In the control group, the BF and GaM muscles followed the same muscle activity pattern from pre to post-test, but the TA and the RF muscle changed the activity pattern from pre to post-test.

### Ergo-jump variable

The results of the Univariate ANCOVA test showed no significant difference between the two groups in TPP (F_(1,17)_ = 0.974, p = 0.337, η^2^ = 0.054), TCP (F_(1,17)_ = 0.676, p = 0.422, η^2^ = 038), TFP (F_(1,17)_ = 0.293, p = 0.595, η^2^ = 017), and power (F_(1,17)_ = 0.114, p = 0.740, η^2^ = 007), whilst adjusting for the pre-test.

## Discussion

This study examined the influence of a 4-week plyometric training on neuromuscular and Ergo-jump characteristics' in different phases of a one-leg drop jump in active girls. The present study showed that the TTP in the plyometric training group compared to the control group in the PR phase showed a significant decrease in the RF muscle. The TTP of the RF muscle in the PR phase, after 4 weeks of plyometric training, decreased by 18% compared to the control group. It means that after training, the RF muscle reduced the reaction time of muscle contraction. Previous studies, such as Chang et al. showed that the time to peak decreases after plyometric training in children^36^. Wu et al. suggested that after plyometric training, muscle activation was enhanced and activated earlier^37^. Also, Kubo et al. reported that plyometric training increased voluntary muscle activation^38^. This result revealed that plyometric training could alter neuromotor control^39^. As a result, the earlier activity of the RF muscle as the main responsible for maintaining the knee joint against additional loads^15^ and the main power transmitter from the thigh to the knee in jumps^40^ helps to stabilize the joint prior to landing. Preparatory muscle activity involves feed-forward processing, in which the planning of movements is based on sensory input from previous experiences^41^. Also, the TA (− 15%), GaM (− 19%), and BF muscle (− 9%) started their activity earlier after plyometric training. That is, by activating the muscles before landing (PR phase), the neuromuscular system prepares the joints for shock absorption^42,43^. Michael et al. showed Anticipation modulates neuromechanics of drop jumps in known or unknown ground stiffness^44^. Also, Guerrero showed that Jumping from a sand surface requires more output from the quadriceps muscles than jumping from a rigid surface^45^. Despite this, other neuromuscular characteristics, such as maximum activity and signal amplitude, did not differ significantly between the two groups in different phases of one leg drop jump. In contrast to the current findings, other studies showed a significant difference after plyometric training. Hammami et al. also noted that an 8-week plyometric program significantly improved the RMS values for the Rectus Femoris in elite soccer players^46^. Similarly, Toumi et al. found significant improvement in RMS values for the knee extensor muscles during SJ after 8 weeks of PT^47^. On the other hand, Mehdipour et al. saw no effect on RMS for the Rectus Femoris and right thigh muscles after 6 weeks of PT^48^. The major part of the improvements during the initial weeks in ballistic type is probably due to adaptations of the neural system, such as increased motor unit firing rate, synchronization, and excitability, an increase in efferent motor drive, and improved co-activation of the synergist muscles^49,50^. These disparate results may reflect differences in the type and intensity of programs and the height of the jumps undertaken.

Also, the results of this research showed that there was no significant difference between the two groups at the Onset time. Despite this, the Onset time decreased from pre to post-training in both groups. It was also shown in the present study that the Activity Time differs between the two groups in the TA muscle. The results of other studies suggest that female athletes have a higher risk of ACL injury during jump landing due to increased TA translation force with quadriceps muscle activity^51^. So plyometric training for reduced injury during jump landing change muscle activity patterns. In plyometric training, the TA and RF muscle followed the same muscle activity pattern from pre to post-test, but the BF and the GaM muscle changed the activity pattern from pre to post-test. The previous studies showed that Muscle-Activation Strategies could change by Plyometric training, sex, and fatigue^2,5,6^. Nicole et al. showed that change Muscle-Activation Strategies represent preprogrammed motor strategies learned during plyometric training^5^. Katsikari et al. reported Kinetic and Kinematic Changes in Prepubescent Girls after Plyometric Training^52^. Lephart et al. showed that plyometric programs may further improve muscular activation patterns^53^. In the control group, the BF and GaM muscle followed the same muscle activity pattern from pre to post-test, but the TA and the RF muscle changed the activity pattern from pre to post-test. In the control group, in the pre-test, as in the training group, TA muscle was used at first. Despite this, it seems that after the subjects learned in the pre-test, the RF muscle was used initially because it has been shown that this muscle can effectively help to stabilize the joint^15^.

The research results showed no significant difference between the two groups in Ergo-jump variables. In contrast, other studies have shown that Plyometric training improves performance and maintains explosive strength parameters^54,55^. Villarreal et al. (2011) report improvements in Power Output, Maximum Rate of Force Development, and Height in Countermovement Jump after plyometric training^56^. The performance of plyometric training always depends on different factors such as training level, gender, age, sports activity, and years of training experience also some other important factors depend upon the plyometric training performance like the type of training, training load, and intensity, duration of the training, and also rest between the repetition and the set^57^. However, a greater jumping performance may not necessarily coincide with sEMG activity conversely^58^.

Results of the current research showed that using the plyometric training in female training programs reduces the maximum landing forces and leads to fewer ACL injuries^42^. Plyometric training also increases joint stability and reduces injury risks by facilitating neuromuscular adaptations^7^. With this study, it may be possible to create this mindset for coaches that plyometric training via changing the muscle activity patterns before landing may result in more stability in the knee joint and decrease the risk of ACL injuries in active women.

### Limitations

The current investigation was conducted on adult females, and there is a need to extend these results to cover other age and gender groups and other skill levels. The study design (cross-sectional) does not allow inferring cause-effect relationships. Therefore, extrapolation of current findings to training interventions should be performed with caution. An extension of the duration of the plyometric program and an increase in exercise intensity might have enabled participants to achieve significant improvements in neuromuscular characteristics and Ergo-jump.

## Conclusion

In conclusion, a 4-week plyometric training made 4 important-lower-limb muscles activated earlier in the preparatory phase compared to the control group which can indicate positive changes in neuromotor control and leads to a safer landing and better stabilization in the knee joint. Additionally, Rectus Femoris and Tibialis Anterior showed the same muscle activity pattern in the training group while they were different in the control group. These findings suggest that plyometric training via changing the muscle activity pattern before landing may result in more stability in the knee joint and decrease the risk of ACL injuries in active women.

## Data Availability

All data generated or analyzed during this study are included in this published article.
